# Cross-Frequency Context-Guided Mamba Network for Infrared Small Target Detection

**DOI:** 10.3390/s26144369

**Published:** 2026-07-09

**Authors:** Hongxin Li, Nan Li, Lin Tian

**Affiliations:** 1 School of Electronic Engineering, Yili Normal University, Yining 835000, China; 20240050188@ylnu.edu.cn (H.L.); 20240050186@ylnu.edu.cn (N.L.); 2 Laboratory of Intelligent Signal Interception and New-Generation Communication Technology, Yili Normal University, Yining 835000, China

**Keywords:** infrared small target detection, Mamba, cross-frequency modeling, context-aware gating, false alarm suppression

## Abstract

Infrared small target detection remains challenging because small targets, cloud edges, sea reflections, building heat sources, and sensor noise often share similar high-frequency responses. Existing local enhancement methods tend to amplify target-like clutter, whereas global modeling methods may dilute sparse target details. To address this issue, this paper proposes CFGMNet (Cross-Frequency Context-Guided Mamba Network), which reformulates infrared small target detection as a high-frequency candidate verification problem constrained by low-frequency background context. Specifically, the proposed CFG-Mamba module decomposes deep features into low-frequency background components and high-frequency candidate regions. Mamba is applied only to the low-frequency branch to capture long-range background dependencies, and the resulting contextual representation is used to gate high-frequency responses, thereby suppressing target-like clutter without indiscriminately enhancing local details. Furthermore, a local contrast gate, a residual attention decoder, and a decoupled prediction head are introduced to perform local saliency calibration, skip-connection noise filtering, and response verification. Experiments on NUAA-SIRST, NUDT-SIRST, and IRSTD-1K demonstrate that CFGMNet achieves a favorable balance between segmentation accuracy, false alarm suppression, and inference efficiency. In particular, CFGMNet achieves 85.27% mIoU and 93.77% F1 on NUAA-SIRST and obtains the lowest false alarm rate on IRSTD-1K, while reaching 141 FPS under forward-pass-only evaluation on an RTX 4090 GPU.

## 1. Introduction

Infrared small target detection aims to accurately segment extremely small targets with limited textural information and low contrast from complex infrared scenes, and holds significant practical value in fields such as maritime search and rescue, early warning surveillance, precision guidance, and intelligent security. Unlike conventional semantic segmentation tasks, small infrared targets typically occupy only a tiny fraction of the entire image. They lack stable shapes, edges, and semantic structural features, and are easily confused with background noise such as cloud edges, sea surface reflections, building heat sources, and sensor noise. Therefore, the key challenge in infrared small target detection is how to enhance the response of genuine small targets while suppressing target-like interference in complex backgrounds.

In recent years, deep learning methods have significantly improved the performance of small target detection in infrared imagery. U-Net-style encoder–decoder architectures have mitigated the loss of small target details through multi-scale feature fusion [1]; long-range modeling methods such as attention mechanisms, Transformers, and Mamba have further enhanced the network’s ability to perceive global context and complex background structures [2,3,4]. However, existing methods still have certain limitations. Although local contrast enhancement or high-frequency feature enhancement methods can highlight small target details, they also tend to amplify bright noise, edge textures, and isolated heat sources in the background; conversely, global modeling methods that directly act on overall features may, while capturing long-range dependencies, amplify false background responses and even weaken the sparse details of small targets.

A key reason for the aforementioned issues is that existing global-local fusion methods largely rely on feature concatenation, weighted fusion, or simple interaction, without fully accounting for the differences in frequency components among targets, backgrounds, and noise in infrared images [5]. Typically, large-scale background structures tend to manifest as low-frequency, smooth components, whereas small targets, background edges, and isolated noise often collectively exhibit localized high-frequency abrupt responses. If feature enhancement is performed solely in the spatial dimension, the model struggles to distinguish between the high-frequency components of real targets and those of background noise. Therefore, establishing a constraint relationship between low-frequency background context and high-frequency candidate regions is key to reducing the false alarm rate and improving detection accuracy.

To address the aforementioned issues, this paper proposes CFGMNet (Cross-Frequency Context-Guided Mamba Network), a cross-frequency context-guided Mamba network designed for infrared small target detection. Unlike methods that directly enhance local saliency responses, this paper models infrared small target detection as a process of validating high-frequency candidate regions under the constraints of low-frequency background context. This method utilizes Mamba [3] to model the long-range dependencies of low-frequency background structures and selectively filters high-frequency candidate regions based on background context. This approach prevents the indiscriminate enhancement of both real small targets and background noise, thereby improving false-alarm suppression performance in complex backgrounds.

Under this framework, this paper further proposes a false alarm detection framework comprising cross-frequency candidate screening, local response calibration, decoding noise filtering, and output response verification. It also incorporates a hard negative sample optimization strategy to enhance the model’s ability to distinguish target signals from background interference.

Crucially, from an engineering perspective, minimizing the false alarm rate is of paramount importance for single-frame detectors. In real-world deployed systems, suppressing target-like clutter to maintain a low Fa takes precedence over slightly higher pixel-level intersection-over-union IoU, as excessive false alarms will trigger frequent system misreports and severely degrade operational reliability.

The main contributions of this paper are summarized as follows:

1. We propose a cross-frequency candidate verification paradigm for low-false-alarm infrared small target detection. Instead of directly enhancing local saliency responses, the proposed paradigm verifies high-frequency candidate regions under the constraint of low-frequency background context.

2. We design a CFG-Mamba module that applies Mamba to the low-frequency background branch rather than the entire feature map. The modeled long-range background context is used to generate spatial gating weights for high-frequency candidate regions, thereby reducing the indiscriminate amplification of target-like clutter.

3. We develop a collaborative low-false-alarm optimization framework that integrates local contrast calibration, residual attention decoding, decoupled target-presence prediction, and hard negative mining. Extensive experiments on three public infrared small target datasets demonstrate that CFGMNet achieves competitive detection accuracy, strong false alarm suppression, and high forward-pass inference efficiency.

## 2. Related Work

### 2.1. Traditional Filtering-Based Infrared Small Target Detection

Early methods for detecting small infrared targets primarily relied on manually designed image priors, achieving target detection through background suppression and target enhancement. These methods generally assume that small infrared targets exhibit characteristics such as abrupt gray-level changes in localized regions, small size, and approximately point-like distribution, whereas the background has a relatively smooth or structured spatial distribution. Consequently, researchers often employ methods such as the Top-hat transform [6], local contrast measurement [7], multiscale relative local contrast measurement [8], weighted local difference measurement [9], max-mean/max-median filtering [10], and multiscale patch-based contrast measurement [11] to suppress the background and highlight target responses.

Traditional filtering methods offer advantages such as simple structure, low computational complexity, and minimal training data requirements, and can achieve reasonable results in scenarios with relatively smooth backgrounds and high target contrast. However, real-world infrared scenes often contain complex background interferences such as cloud cover, sea surface ripples, building edges, tree textures, and sensor noise. These areas may also exhibit strong local contrast or high-frequency responses, making them prone to being misclassified as targets by traditional filtering methods, thereby resulting in a high false alarm rate. Furthermore, traditional methods typically rely on fixed windows, fixed scales, or manually set thresholds, making it difficult for them to adapt to variations in target size, imaging conditions, and background complexity. Consequently, under conditions involving small targets, low signal-to-noise ratios, and complex backgrounds, the generalization capability and robustness of traditional filtering methods remain limited.

### 2.2. CNN-Based Infrared Small Target Detection

With the advancement of deep learning, infrared small target detection methods based on convolutional neural networks have gradually become mainstream. Representative CNN-based methods include ACM [12], DNANet [13], UIU-Net [14], ALCNet [15], and attention-guided pyramid context networks [16]. These methods enhance small target representation through contextual modulation, dense feature fusion, nested U-shaped structures, local contrast attention, or pyramid context modeling. Recent surveys and related studies have further summarized the development of infrared small target segmentation networks and attention-based detection methods [17,18,19]. In addition, improved U-Net and dense nested U-Net variants have also been developed to enhance multi-scale feature decomposition, feature fusion, and background suppression [20,21].

Although CNN-based methods offer stronger feature representation capabilities than traditional methods, convolutional operations primarily focus on local neighborhood information, making it difficult to adequately model long-range background dependencies and global scene context. For small infrared targets lacking distinct texture and semantic information, bright noise, edge textures, and local anomalies in complex backgrounds are still prone to being misidentified as targets. Although multi-scale fusion and attention mechanisms can enhance target responses [19], they may simultaneously amplify false target regions in the background, leading to an increased false alarm rate. Therefore, how to introduce more effective global contextual constraints while retaining the CNN’s advantage in modeling local details has become an important direction for future research.

### 2.3. Transformer- and State-Space-Model-Based Infrared Small Target Detection

To address the limitations of CNNs in modeling long-range dependencies, Transformers have gradually been introduced into infrared small target detection tasks. By leveraging self-attention mechanisms, Transformers can establish feature correlations over a wider range, thereby enhancing the model’s ability to perceive complex background structures and long-range contextual information. Existing methods typically employ hierarchical visual Transformers, CNN-Transformer hybrid architectures, or Swin Transformer modules to improve the discrimination of small, weak targets in complex backgrounds. For example, IRSTFormer models multi-scale contextual information through a hierarchical Transformer [22], and related Transformer methods have also been applied to the detection of small and dim infrared targets under complex backgrounds [23].

However, the self-attention mechanism of Transformers typically involves high computational complexity, resulting in significant computational overhead in the processing of high-resolution infrared images. Additionally, since small infrared targets have very small areas and sparse textures, their weak responses are easily diluted by complex background information during global modeling, leading to missed detections. To address these issues, the state-space model Mamba has gradually been applied to the detection of small infrared targets due to its linear computational complexity and efficient long-range modeling capabilities. More recently, Mamba-based methods have attracted attention because of their linear computational complexity and efficient long-range modeling capability. Representative methods include MiM-ISTD [24], IRMamba [25], SAMamba [26], and EAMNet [27]. In addition, recent spatial-frequency representation learning methods further indicate the importance of frequency-aware modeling for infrared small object detection [28].

The essential differences between CFGMNet and representative Transformer- and Mamba-based methods are summarized in Table 1.

## 3. Methodology

**Figure 1:**
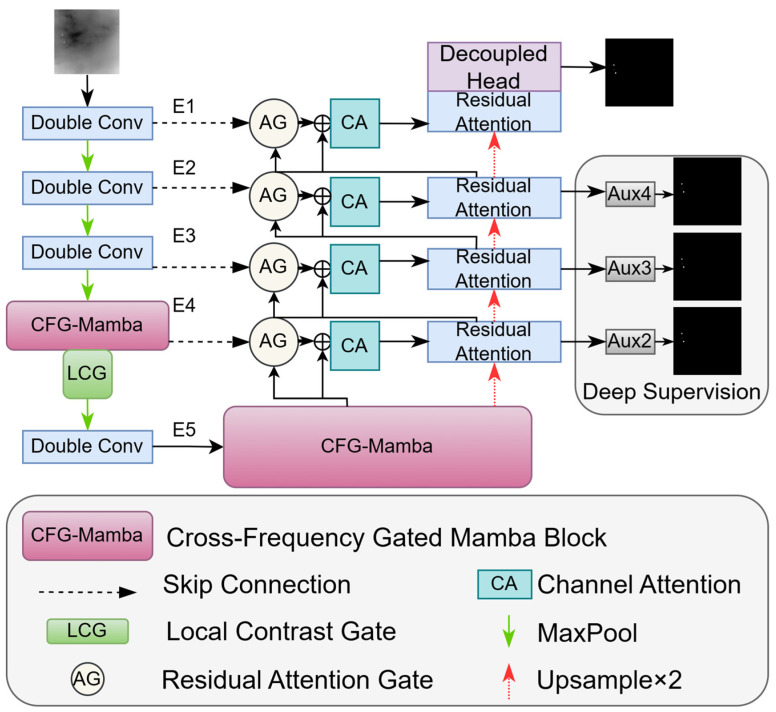
Overall architecture of the proposed CFGMNet. The network consists of a multi-scale encoder, two CFG-Mamba modules, a Local Contrast Gate, a residual attention decoder, and a decoupled prediction head. All components are trained from scratch without pre-trained weights or frozen layers.

To improve the accuracy of small target detection in complex infrared backgrounds and reduce false alarms caused by factors such as local bright clutter, cloud edges, sea surface reflections, and building heat sources. Specifically, it first uses low-frequency background context to constrain high-frequency local responses, and then further suppresses false target responses through local contrast calibration and decoupled prediction. The overall architecture is shown in Figure 1.

Given a single-channel infrared image I, the network first extracts shallow, basic features using an initial dual-convolution unit, and then feeds them into a multi-scale convolutional encoder. The encoder generates encoded features at different scales through successive downsampling, and its recursive process can be expressed as follows:

(1) $$E_{i}=\varepsilon_{i}(E_{i-1}),\;\;\;\;\;\;\;\;\;\;\;i=1,2,\ldots ,L$$

where Ei denotes the features encoded in layer i, where $\varepsilon_{i}$ represents the corresponding encoding unit. As the network progresses to deeper layers, feature resolution gradually decreases while the number of channels gradually increases. Shallow-layer features retain more spatial details, whereas deep-layer features possess stronger semantic representation capabilities and larger receptive fields.

To address the issue that local enhancement tends to amplify background noise, this paper introduces the CFG-Mamba module at the deep encoding stage to perform context-constrained filtering of candidate target responses.

Following cross-frequency filtering, this paper further introduces the Local Contrast Gate (LCG) module to calibrate the local saliency of the candidate region. By estimating the local average background and applying forward differential responses, the LCG further suppresses isolated thermal noise and locally anomalous high-brightness regions. The processed features are downsampled to obtain bottleneck features, which are then processed again by CFG-Mamba to enhance global context modeling capabilities.

Spatial resolution is restored through successive upsampling during the decoding stage. To prevent shallow skip connections from directly feeding background textures and highlight noise into the decoder, this paper uses upsampled decoding features as semantic guidance to perform residual attention filtering on encoded features at the corresponding scale. These features are then concatenated with the upsampled features, and adaptive weighting is achieved through channel attention:

(2) $$D_{i}=D_{i}(C_{i}([G_{i},{\tilde{E}}_{i}]))$$

where $G_{i}$ denotes the upsampled decoded features, ${\tilde{E}}_{i}$ denotes the skip-connection features filtered by residual attention, and $C_{i}$ and $D_{i}$ denote the channel attention and decoding convolutional units, respectively.

The output layer uses a dual-branch decoupled prediction head, which predicts the target mask $P_{\mathrm{mask}}$ and the target presence confidence $P_{\mathrm{obj}}$ separately. During the inference phase, the outputs from the two branches are multiplied element-wise after passing through a Sigmoid activation function to obtain the final prediction:

(3) $$P=\sigma (P_{\mathrm{mask}})⊙\sigma (P_{\mathrm{obj}})$$

This fusion method acts as a soft “AND” gate mechanism, retaining only those locations that simultaneously exhibit pixel-level target responses and high confidence in target presence. During training, multi-scale deep supervision is introduced to enhance the ability to localize small targets at different decoding levels; during inference, only the main output is retained, avoiding additional computational overhead. Overall, CFGMNet establishes a coarse-to-fine low-false-alarm-rate infrared small target detection workflow through cross-scale context filtering, local contrast calibration, skip-connection noise filtering, and decoupled prediction validation.

### 3.1. Cross-Frequency Gated Mamba Module

**Figure 2:**
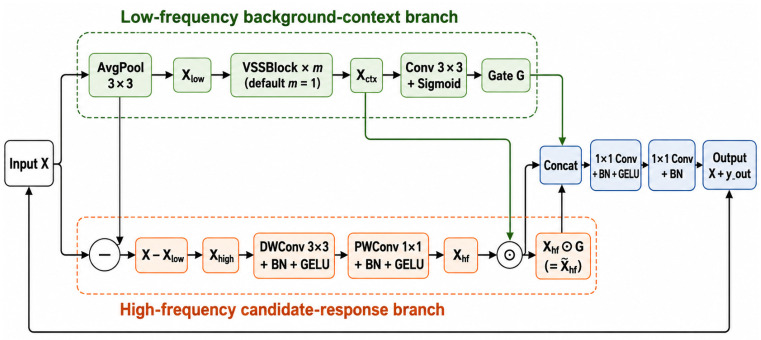
Structure of the Cross-Frequency Gated Mamba module. The input feature is decomposed into low-frequency background context and high-frequency candidate responses. The low-frequency branch generates a spatial gate to selectively verify high-frequency target-like responses.

The CFG-Mamba module explicitly assigns different functional roles to different frequency components of deep features, as illustrated in Figure 2. The low-frequency component, obtained by average pooling, is dominated by smooth background structures and is processed by the Mamba-based low-frequency branch to capture long-range background dependencies. The high-frequency component, computed as the residual between the original feature and the low-frequency component, is dominated by local abrupt responses, including true small target cues, background edges, highlight clutter, and sensor noise, and is processed by a lightweight local transformation. Cross-frequency filtering is performed when the low-frequency contextual feature generates a spatial gating map to modulate the high-frequency candidate region. Therefore, the interaction between different frequency components is not implemented by simple concatenation, but by an asymmetric low-to-high-frequency gating mechanism.

Given an input feature map $X\in {\mathbb{R}}^{B\times C\times H\times W}$, the low-frequency component $X_{\mathrm{lf}}$ is obtained by a 3 × 3 average-pooling operation with stride 1 and padding 1, which serves as a lightweight low-pass operation and mainly extracts smooth background-dominated information. The high-frequency component is then computed as the residual between the original feature and the low-frequency $X_{\mathrm{hf}}$ component, which mainly preserves local abrupt variations. In this sense, the average-pooling branch dominates the extraction of low-frequency background context, whereas the residual branch dominates the extraction of high-frequency candidate regions.

(4) $$X_{\mathrm{lf}}=AvgPool_{3\times 3}(X)$$

(5) $$X_{\mathrm{hf}}=X-X_{\mathrm{lf}}$$

Specifically, $X_{\mathrm{lf}}$ mainly contains smoother background structures, while $X_{\mathrm{hf}}$ retains local abrupt responses, including true small target cues, background edges, highlight clutter, and sensor noise. Average pooling is adopted because it is parameter-free, computationally lightweight, and easy to integrate into the network. Alternative low-pass operations are further analyzed in Section 4.5.4.

The low-frequency branch is modeled by a Visual State Space Block (VSSBlock). Given $X_{\mathrm{lf}}$ the feature map is first flattened into a one-dimensional sequence in row-major order:

(6) $$X_{\mathrm{seq}}=F\mathrm{latten}(X_{\mathrm{lf}})\;X\in {\mathbb{R}}^{L\times C},L=H\times W$$

After sequence serialization, LayerNorm, Mamba modeling, and reshaping, the modeled low-frequency feature is fused with the original low-frequency component through a residual connection:

(7) $$X_{ct}=X_{\mathrm{lf}}+\alpha \cdot VSSB\mathrm{lock}(X_{\mathrm{lf}})$$

**Figure 3:**
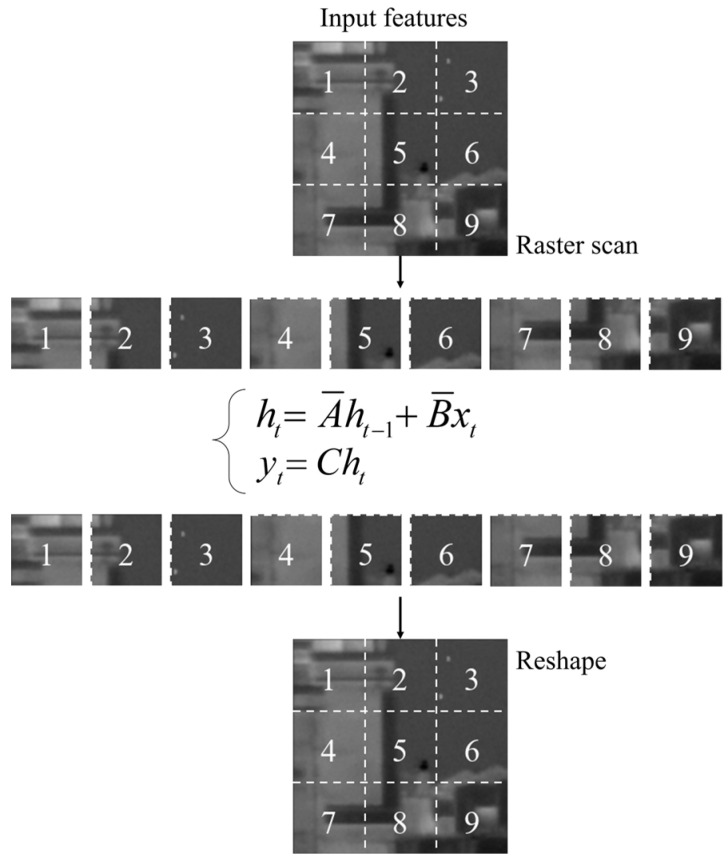
Sequence modeling process of the VSSBlock. The low-frequency feature map is serialized in row-major order, processed by LayerNorm and Mamba, and then reshaped back into a two-dimensional contextual feature map.

The process is shown in Figure 3.

In the default configuration, each CFG-Mamba module contains one VSSBlock, mamba_depth=1. The Mamba layer uses a model dimension equal to the input channel number, a state dimension of 16, an internal convolution width of 4, and an expansion factor of 2. In the complete network, two CFG-Mamba modules are deployed. The first one is inserted after the fourth encoder stage and followed by the Local Contrast Gate, while the second one is placed at the bottleneck stage. For an input image of 256×256 and a base channel number of 48, the corresponding feature dimensions of the two CFG-Mamba modules are 32×32×384 and 16×16×768, respectively. Therefore, the complete network contains two VSSBlocks in total.

Where $X_{ct}$ denotes the low-frequency contextual feature, and α is a learnable residual scaling parameter. Therefore, $X_{ct}$ contains both the original low-frequency background structure and the long-range contextual dependencies modeled by Mamba.

Next, a spatial gating map is generated from the low-frequency contextual feature:

(8) $$G=\sigma (\mathrm{Conv}3\times 3(X_{ct}))$$

where σ (·) denotes the Sigmoid activation function, and $G\in {\left[0,1\right]}^{1\times H\times W}$ represents the spatial response weights under background-context constraints. The VSSBlock is embedded within the low-frequency branch of the CFG-Mamba module.

For the high-frequency branch, a lightweight local transformation is applied to $X_{\mathrm{hf}}$ to obtain high-frequency candidate regions:

(9) $${\hat{X}}_{\mathrm{hf}}=\phi (X_{\mathrm{hf}})$$

where ϕ(⋅) consists of a depthwise 3 × 3 Conv-BN-GELU layer followed by a pointwise 1 × 1 Conv-BN-GELU layer. This design preserves local high-frequency candidate regions while introducing lightweight channel interaction.

The cross-frequency filtering is then explicitly performed by using the low-frequency context-guided gate to modulate the high-frequency candidate regions:

(10) $$X_{g}=G⊙{\hat{X}}_{\mathrm{hf}}$$

where ⊙ denotes element-wise multiplication, and $X_{g}$ denotes the gated high-frequency feature. This operation indicates that cross-frequency interaction occurs at the gating step: the low-frequency contextual branch provides background-aware spatial weights, while the high-frequency branch provides candidate target responses. Therefore, the high-frequency responses are not directly enhanced, but are selectively verified under the constraint of low-frequency background context.

Finally, the low-frequency contextual feature and the gated high-frequency feature are concatenated along the channel dimension and fused by two successive 1 × 1 convolutional layers:

(11) $$Y=X+\beta \cdot f([X_{ct},{\tilde{X}}_{\mathrm{hf}}])$$

where [⋅,⋅] denotes channel-wise concatenation, f(⋅) denotes the fusion operation composed of 1 × 1 Conv-BN-GELU followed by 1 × 1 Conv-BN, and β is a learnable residual scaling parameter. Through this design, CFG-Mamba provides context-constrained candidate filtering for subsequent local contrast calibration and decoding prediction.

### 3.2. Local Contrast Gating Module

**Figure 4:**
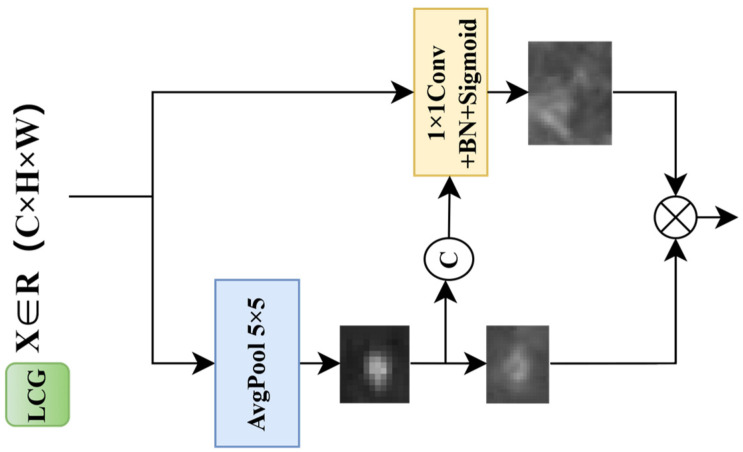
Structure of the Local Contrast Gate module. The module estimates local background responses and generates a contrast-aware spatial gate to suppress isolated background noise while preserving salient target candidates.

The LCG module performs neighborhood-level saliency calibration on the filtered features, with its structure shown in Figure 4. It constructs local saliency constraints by estimating per-pixel neighborhood background in the feature space.

Given the input feature $X\in {\mathbb{R}}^{B\times C\times H\times W}$, the module first estimates the local background of each pixel using average pooling with a 5 × 5 window, a stride of 1, and a padding of 2:

(12) $$B_{\mathrm{loc}}={\mathrm{AvgPool}}_{5\times 5,s=1,p=2}(X)$$

The 5 × 5 window is adopted as an empirical trade-off between local background estimation and small target response preservation, rather than as a strict assumption about a fixed target size. A smaller window may provide insufficient neighborhood context, whereas a larger window may introduce excessive background information and suppress weak target responses, as further analyzed in the receptive-field ablation study.

Next, the difference between the original features and the estimated local background is calculated, and the regions with positive local responses are retained using the ReLU activation function to obtain the local contrast difference features:

(13) $$D_{\mathrm{loc}}=ReLU(X-B_{\mathrm{loc}})$$

Infrared small targets in commonly used IRSTD benchmarks usually appear as locally salient responses relative to their surrounding background. Therefore, the proposed LCG enhances positive local deviations while implicitly suppressing low-contrast background fluctuations in the learned feature space and should be interpreted as a local saliency calibration mechanism rather than an absolute image-level assumption that all targets must be brighter than the background. This design helps suppress isolated local anomalies while preserving candidate regions with distinguishable contrast. Nevertheless, the current LCG mainly focuses on positive local contrast, and its applicability to extremely dark-target or contrast-reversed scenarios remains limited. Bidirectional contrast modeling and adaptive local contrast estimation will be explored in future work.

Concatenate the original features with the contrast difference features along the channel dimension, and generate an adaptive spatial gating map using 1 × 1 convolutions, batch normalization, and the Sigmoid activation function:

(14) $$G_{\mathrm{loc}}=\sigma (BN({\mathrm{Conv}}_{1\times 1}([X;D_{\mathrm{loc}}])))$$

The thresholding map combines both the semantic information of features and local contrast information; a region is assigned a higher weight only when it possesses both the semantic features of the target and satisfies the local brightness contrast conditions. Finally, the gated map is multiplied element-wise with the original features to obtain the filtered output features:

(15) $$Y_{\mathrm{lcg}}=X⊙G_{\mathrm{loc}}$$

### 3.3. Residual Attention Decoder

**Figure 5:**
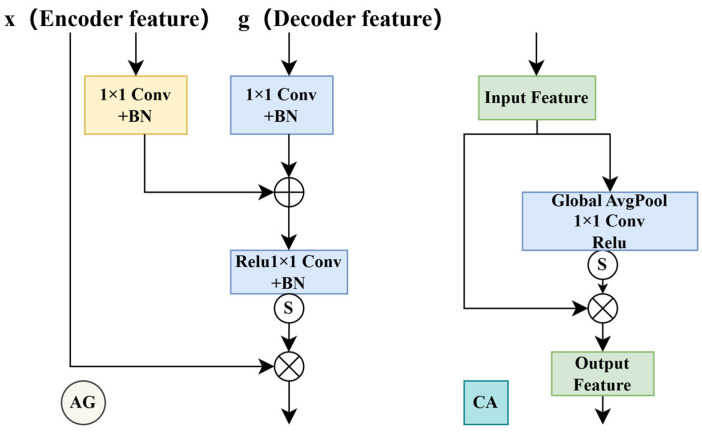
Structure of the residual attention decoder. Decoder features guide the filtering of encoder skip-connection features, and residual channel attention further recalibrates the fused features to reduce background clutter.

This module performs spatial filtering on skip-connection features and channel recalibration on fused features, as shown in Figure 5. The residual design follows the idea of residual learning [29], while the spatial attention gate and channel recalibration are inspired by attention-gated U-shaped networks [30] and squeeze-and-excitation channel attention [31], respectively.

At each decoding stage, the decoded features from the previous layer are first bilinearly upsampled to obtain the decoded features g at the current scale. Simultaneously, the corresponding skip-connection features x are retrieved from the encoder. Here, g contains stronger deep semantic information and can be used to provide target-relevant contextual guidance; x contains richer spatial details but also includes a significant amount of background noise. Therefore, this paper employs an attention gate to filter x using g, rather than directly concatenating x with g.

Specifically, the decoder feature $g_{i}$ and the encoder feature $x_{i}$ undergo channel mapping via 1 × 1 convolutions and BatchNorm, respectively, so that both are projected onto the same intermediate feature dimension. Subsequently, the two feature streams are added together and passed through a ReLU activation, a 1 × 1 convolution, and a Sigmoid function to obtain the spatial attention weights $\psi_{i}$. These weights reflect the consistency between the shallow-level details of the encoder and the deep-level semantics of the decoder, and are used to determine which skip-connection regions should be preserved:

(16) $$\psi_{i}=\sigma (Conv1\times 1(\mathrm{ReLU}(P_{x}(x_{i})+P_{g}(g_{i}))))$$

(17) $${\tilde{x}}_{i}=[1+\gamma s(2\psi_{i}-1)]⊙x_{i}$$

where ψ is constrained in [0, 1] via Sigmoid normalization. Where $x_{i}$ represents the feature from the encoder skip connection, $g_{i}$ represents the upsampled decoder feature, $P_{x}(\cdot )$ and $P_{g}(\cdot )$ denote the 1 × 1 convolutional projection operations for the encoder and decoder features, respectively, $\psi_{i}$ represents the spatial weights generated by the attention gate, and $\gamma_{s}$ is a learnable spatial residual scaling parameter. The term 2 $\psi_{i}$ −1 maps the attention weights from [0, 1] to [−1, 1], enabling the module to suppress low-attention background responses while enhancing high-attention target-related responses. Since $\gamma_{s}$ is initialized with a small value, this module approximates an identity mapping in the early stages of training and does not disrupt the information flow of the original U-Net skip connection. As training progresses, the model can gradually learn to enhance regions relevant to the target while suppressing background textures and noise.

The AG-filtered skip connection feature ${\tilde{x}}_{i}$ and the upsampled decoded feature $g_{i}$ are concatenated along the channel dimension:

(18) $$F_{i}=Concat({\tilde{x}}_{i},g_{i})$$

Subsequently, this paper further employs residual channel attention to recalibrate the fused feature $F_{i}$. Channel attention first extracts channel-level statistical information via global average pooling, then generates channel weights through two 1 × 1 convolutions followed by a Sigmoid function. This process adaptively adjusts the importance of different channels, enhancing those associated with small target responses while attenuating the influence of redundant background channels. Its residual form is:

(19) $$s_{i}=\sigma (Conv_{1\times 1}(\delta (Conv_{1\times 1}(GAP(F_{i})))))$$

(20) $${\tilde{F}}_{i}=F_{i}+\gamma_{c}(s_{i}⊙F_{i})$$

where $s_{i}$ represents the channel attention weights, δ(⋅) denotes the nonlinear activation function, and $\gamma_{c}$ is a learnable channel residual scaling parameter. Finally, the feature ${\tilde{F}}_{i}$, which has undergone spatial filtering and channel recalibration, is fed into the dual convolutional module to generate the output feature for the current decoding layer. As a result, the residual attention decoding module is able to retain spatial localization information from earlier layers while reducing the risk of background noise introduced by skip connections.

### 3.4. Decoupling the Prediction Head

**Figure 6:**
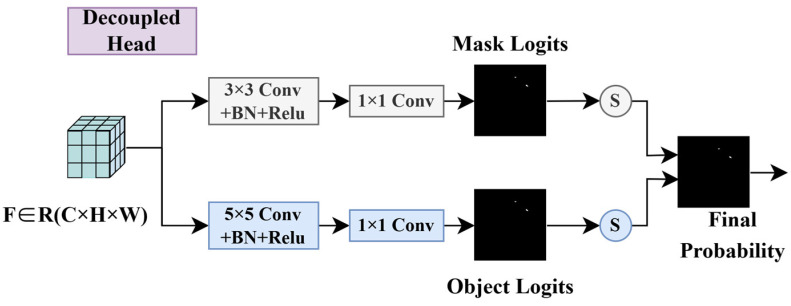
Structure of the decoupled prediction head. The Mask branch predicts pixel-level target regions, while the Object branch verifies target presence. Their Sigmoid outputs are multiplied during inference to suppress isolated false responses.

The final prediction is decoupled into two parallel sub-tasks: pixel-level target mask prediction and regional target presence determination. The Mask branch is responsible for pixel-level position and shape prediction, while the Object branch determines whether candidate regions correspond to real targets. During the inference stage, the outputs from the two branches are fused via multiplicative fusion to form a soft consistency constraint, thereby suppressing isolated false responses that lack support from the object presence. The structure of the decoupled prediction head is shown in Figure 6.

This module takes the top-level output features from the decoder as input and divides them into a Mask branch and an Object branch in parallel. The Mask branch uses 3 × 3 convolutions to extract local detail features and is primarily responsible for predicting the shape, boundaries, and pixel-level location of the object; the Object branch uses 5 × 5 convolutions to introduce a larger receptive field and is primarily responsible for determining whether a real object exists in the current response region. After both branches undergo batch normalization and ReLU activation, they are mapped to single-channel outputs via 1 × 1 convolutions, yielding the object mask logits $P_{mask}$ and the object presence logits $P_{obj}$, respectively.

Where $P_{mask}$ represents the segmentation confidence that a pixel belongs to the target region, and Pobj represents the confidence that a true object exists in that region. During the inference stage, the final predicted probability map P is obtained by multiplying the Sigmoid-activated outputs of these two branches element-wise, as previously defined in Equation (3). This fusion mechanism acts as an “AND gate”; a region is classified as a target only when it simultaneously satisfies both conditions—“shape matches the characteristics of a small target” and “is semantically a true target”—effectively filtering out background noise points that are similar in shape but semantically mismatched. For false activations caused by isolated noise points, background edges, or local texture changes, even if they generate a high response in the Mask branch, the final output will be effectively suppressed as long as the Object branch determines that they lack the attributes of a real target. Consequently, by leveraging the multiplicative constraints between the Mask branch and the Object branch, the decoupled prediction head enhances the output’s ability to filter out pseudo-responses with minimal computational overhead.

### 3.5. Loss Function

To address the issues of extreme positive-to-negative sample imbalance, low target pixel coverage, and false positives caused by complex backgrounds in small target infrared detection, this paper proposes a composite loss function optimized for low false positive rates. This loss function combines OHEM-BCE, Dice Loss [32], Soft IoU Loss, and Focal Loss [33] to constrain model training in three aspects: difficult negative sample mining, region overlap optimization, and class imbalance suppression.

First, Online Hard Example Mining Binary Cross Entropy (OHEM-BCE) is used to select difficult negative samples with high loss for inclusion in the optimization process in real time, while ignoring a large number of easily classified background pixels. This allows the model to focus on learning the background regions most likely to be misclassified as objects. Given the functional differences between the prediction branches, the Mask branch focuses on predicting object shapes and pixel-level locations, while the Object branch focuses on object presence classification. Therefore, this paper applies differentiated hard example constraints to the different branches to enhance the model’s ability to suppress high-luminance noise, background edges, and object-like clutter.

Second, Dice Loss and Soft IoU Loss optimize the alignment between predicted results and ground-truth labels from the perspectives of region overlap and intersection-over-union, respectively. Since small infrared targets typically occupy only a tiny fraction of the image, relying solely on pixel-level cross-entropy is prone to being dominated by a large number of background pixels. In contrast, Dice Loss and Soft IoU Loss are more sensitive to target regions, effectively improving the completeness and localization accuracy of small target regions. Furthermore, Focal Loss mitigates the severe imbalance between foreground and background by using a modulation factor to reduce the weight of easily classified samples and increase the contribution of hard-to-classify samples. In this paper, we set its balancing parameters α = 0.75, and focusing parameter = 2.

To address the dual-branch structure of the decoupled prediction head, this paper employs a differential loss supervision strategy. The Mask branch is responsible for predicting object shapes, edges, and pixel-level regions, and uses a weighted combination of four loss functions for supervision:

(21) $$L_{mask}=L_{OHEM}^{0.05}(Z_{m},Y)+0.25L_{Dice}(Z_{m},Y)+0.25L_{IoU}(Z_{m},Y)+0.5L_{Focal}(Z_{m},Y)$$

Here, $Z_{m}$ represents the logits output by the Mask branch, and Y represents the ground-truth segmentation labels.

The Object branch is responsible for determining the presence of objects; its primary role is to suppress false positives caused by bright background noise. It achieves this by mining a higher proportion of hard negative samples and constraining them using the Dice loss:

(22) $$L_{obj}=L_{OHEM}^{0.10}(Zo,Y)+0.2L_{Dice}(Zo,Y)$$

Here, Zo refers to the logits output by the Object branch. The main output loss is composed of the weighted sum of the losses from the two branches:

(23) $$L_{main}=L_{mask}+0.5L_{obj}$$

To mitigate the problem of gradient vanishing in deep network training, this paper introduces a multi-scale deep supervision mechanism by adding auxiliary output heads to the three intermediate layers of the decoder. Each auxiliary output employs a composite loss function consistent with that of the Mask branch. The total loss is calculated using a pyramid-weighted sum, where the main output is weighted 1.0, and the weights of the three auxiliary outputs decrease with feature scale to 0.5, 0.25, and 0.125, respectively:

(24) $$L_{total}=\frac{\sum_{i=0}^{3}wiLi\sum }{\sum_{i=0}^{3}wi}$$

Here, wi represents the loss weights for outputs at different scales, L0 is the main output loss, and L1 through L3 are the three auxiliary output losses.

During the inference phase, the final predicted probability map is directly obtained by fusing the two decoupled branches using Equation (3).

The loss terms are designed to be complementary rather than redundant. OHEM-BCE performs explicit discrete hard negative selection, while Focal Loss applies continuous difficulty-aware reweighting; their discrete-continuous synergy improves robustness without gradient conflicts. Dice Loss and Soft IoU Loss focus on region-level geometric consistency, which is less sensitive to pixel-wise imbalance. This pixel-region coupling forms a hierarchical supervision mechanism: pixel-level hard negative suppression reduces spurious boundary activations to improve region-level optimization, while region-level constraints stabilize pixel-wise learning under severe imbalance. All loss weights are empirically determined following common practice in dense prediction tasks, and the model maintains stable performance across a wide range of weight perturbations, indicating strong robustness to hyperparameter tuning in complex infrared scenarios.

## 4. Experimental Results and Discussion

### 4.1. Datasets and Evaluation Metrics

The experiment selected three authoritative public datasets in the field of small target infrared detection—NUAA-SIRST, NUDT-SIRST, and IRSTD-1K—covering different scenarios, resolutions, and background complexities [12,34,35]. Each dataset contains 427, 1327, and 1001 infrared images, respectively, with targets primarily consisting of long-range drones, small vessels, and aerial vehicles. Additionally, the SIRST3 dataset, as defined in [4], combines these three datasets to provide a more diverse and comprehensive benchmark for evaluation.

To ensure a fair and reproducible comparison, all experiments follow the official training/testing splits of the corresponding datasets. The baseline results are reproduced based on the publicly available code or implementation details of the corresponding methods. All baseline models are trained and evaluated under the same data splits and evaluation protocol, with the random seed fixed to 42. For CFGMNet, the entire experimental pipeline, including data loading, model initialization, training, and evaluation, is fixed with specified random seeds. Specifically, three independent runs are conducted with random seeds of 42, 123, and 456 to assess the stability of the proposed method. The statistical results across the three runs are reported in Section 4.2.

**Figure 7:**
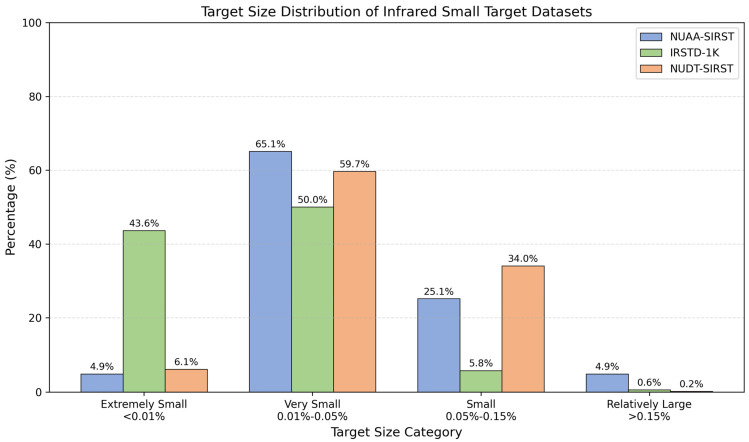
Relative target-size distribution of the three IRSTD datasets. Target size is measured as the ratio between target area and image area, reflecting the scale differences among NUAA-SIRST, NUDT-SIRST, and IRSTD-1K.

To further quantify the differences in target characteristics across the three datasets, we analyzed the relative size distribution of targets in each dataset (the proportion of the target area relative to the entire image), as shown in Figure 7.

The results indicate significant differences in target size distribution among the three datasets: in NUAA-SIRST, 65.1% of the targets are very small targets (0.01–0.05%); In IRSTD-1K, very small targets (<0.01%) account for as much as 43.6%, the highest proportion of small targets among the three datasets; whereas in NUDT-SIRST, relatively large targets (0.05–0.15%) account for 34.0%, far exceeding the proportions in the other two datasets. These differences in scale distribution are a key reason for the varying performance of different methods across the datasets.

During training, we normalized all images and resized them to 256 × 256 for input. The training set underwent data augmentation techniques such as random horizontal flipping, vertical flipping, random cropping, and grayscale normalization to improve the model’s generalization ability. To comprehensively evaluate model performance, we used five mainstream evaluation metrics: mean Intersection-over-Union (mIoU), normalized Intersection-over-Union (nIoU), detection probability (Pd), false alarm rate (Fa), and F1 score. Considering recent discussions on the evaluation reliability of infrared small target detection, we report both pixel-level and target-level metrics, including mIoU, nIoU, Pd, Fa, and F1 [36]. Their definitions are given below.

The mean intersection-over-union (mIoU) is used to measure the overlap between the predicted image and the ground truth label, where $A_{Inter}$ represents the intersection between the predicted result and the ground truth label, and $A_{Union}$ represents the union between the predicted result and the ground truth label. The formula is as follows:

(25) $$mIou=\frac{A_{Inter}}{A_{Union}}=\frac{\sum_{i=1}^{N}\mathrm{TP}[i]}{\sum_{i=1}^{N}\left(T[i]+P[i]-\mathrm{TP}[i]\right)}$$

where N is the number of test images, TP[i] denotes the number of true-positive pixels in the i-th image, T[i] denotes the number of ground-truth positive pixels, and P[i] denotes the number of predicted positive pixels.

The normalized Intersection-over-Union (nIoU) is calculated by averaging the IoU of each image:

(26) $$nIou=\frac{1}{N}\sum_{i=1}^{N}\frac{\mathrm{TP}[i]}{T[i]+P[i]-\mathrm{TP}[i]}$$

Detection probability (Pd) evaluates the target-level detection capability:

(27) $$Pd=\frac{N_{\det}}{N_{gt}}$$

where $N_{\det}$ denotes the number of correctly detected targets and $N_{gt}$ denotes the total number of ground-truth targets.

False alarm rate (Fa) measures the density of false-positive pixels over the evaluated image area:

(28) $$Fa=\frac{P_{\mathrm{fp}}}{P_{\mathrm{all}}}\times 10^{6}$$

where $P_{\mathrm{fp}}$ denotes the number of false-positive pixels and $P_{all}$ denotes the total number of pixels in the evaluated test set. In this paper, Fa is reported in units of $10^{-6}$, namely false-positive pixels per million pixels.

F1 is the harmonic mean of precision and recall:

(29) F1=2×Prec×RecPrec+Rec

(30) Prec=TPTP+FP,Rec=TPTP+FN

TP, FP, and FN denote the numbers of true-positive, false-positive, and false-negative pixels, respectively.

The experiments were conducted on an NVIDIA RTX 4090 GPU platform with 24 GB of video memory, an AMD EPYC 7502 CPU, and 100 GB of system memory. During training, input images were uniformly cropped to 256 × 256, the batch size was set to 8, and the total number of training epochs was 1000. The AdamW optimizer [37] was used with an initial learning rate of 5 × 10^−5^ and a weight decay coefficient of 1 × 10^−4^. The implementation was based on Python 3.8 and PyTorch 2.1.1 [38], with GPU acceleration provided by CUDA 11.8. A learning rate scheduling strategy combining warm-up and cosine decay was employed, with the minimum learning rate set to 1 × 10^−6^. To improve training stability, the gradient clipping threshold was set to 0.5, and the EMA strategy was used to smooth the update of model parameters with a decay factor of 0.999. The loss function employs a combined loss composed of OHEM-BCE, Dice Loss, Soft-IoU Loss, and Focal Loss to mitigate the issues of positive-negative sample imbalance and background false alarms in infrared small target detection. During the evaluation phase, single-scale inference is employed, and the model’s probability map is binarized using a threshold of 0.5. Model performance is ultimately evaluated using metrics such as mIoU, nIoU, F-measure, Pd, and Fa.

### 4.2. Comparison with State-of-the-Art Methods

To comprehensively validate the effectiveness of CFGMNet in the task of small target detection in infrared images, this paper compares it with 10 representative methods on three public benchmark datasets: NUAA-SIRST, NUDT-SIRST, and IRSTD-1K. These include four traditional methods—Top-Hat [6], TLLCM [7], Max-Median [10], and WSLCM [8]—as well as six deep learning methods: ACM [12], ALCNet [15], DNANet [13], UIU-Net [14], SCTransNet [4], and EAMNet [27]. All methods are evaluated using the metrics defined in Section 4.1, including Pd, Fa, F1, mIoU, and nIoU. The quantitative results are shown in Table 2, where bold text indicates the best result and underlined text indicates the second-best result.

To further evaluate the statistical stability of the proposed CFGMNet, we conduct additional experiments under three independent runs with different random seeds (42, 123, and 456). Table 3 reports the mean and standard deviation of all evaluation metrics across the SIRST3 benchmark and three individual datasets.

As shown in Table 3, CFGMNet maintains consistently stable performance across different runs, with very small standard deviations on all metrics, indicating strong robustness against random initialization and training stochasticity. In particular, the variation in mIoU and nIoU remains within a narrow range across all datasets, demonstrating that the proposed cross-frequency modeling and gating mechanism is not sensitive to parameter initialization.

As shown in Table 2, CFGMNet achieves competitive detection performance across all three datasets, with particularly outstanding results on the NUAA-SIRST and IRSTD-1K datasets. On the NUAA-SIRST dataset, CFGMNet achieved a detection probability (Pd) of 99.08%, the highest among all methods; its F1 score, mIoU, and nIoU reached 93.77%, 85.27%, and 86.09%, respectively, all of which are state-of-the-art results. Compared to SCTransNet, CFGMNet improves F1, mIoU, and nIoU by 4.67, 4.95, and 2.49 percentage points, respectively, indicating that our method effectively enhances pixel-level representation capabilities for small target regions. Although CFGMNet has a slightly higher false alarm rate than EAMNet on NUAA-SIRST, 5.98 versus 4.94, it achieves substantially better Pd, F1, mIoU, and nIoU. This indicates that CFGMNet obtains a better overall trade-off between detection completeness and segmentation accuracy.

On the NUDT-SIRST dataset, CFGMNet did not achieve the best results across all metrics. Specifically, UIU-Net achieved the highest Pd, while SCTransNet achieved the lowest Fa as well as the highest F1, mIoU, and nIoU. CFGMNet’s Pd, Fa, F1, mIoU, and nIoU are 95.87%, 9.32 × 10^−6^, 94.29%, 89.18%, and 90.02%, respectively. While it remains highly competitive overall, it falls short of UIU-Net and SCTransNet on certain metrics. This indicates that our method still has room for improvement in scenarios with significant variations in target size, blurred boundaries, or low signal-to-noise ratios. A possible reason is that in such scenarios, the high-frequency differences between real targets and background noise are attenuated, making it more difficult for the cross-frequency gating module to distinguish between target candidates and background false responses; simultaneously, local contrast gating may suppress some true responses when handling targets with blurred boundaries.

On IRSTD-1K, CFGMNet achieves the lowest Fa of 9.11 and slightly outperforms SCTransNet in F1, mIoU, and nIoU. Although the improvements in segmentation metrics are moderate, the simultaneous reduction in false alarms demonstrates the effectiveness of the proposed low-false-alarm design.

**Figure 8:**
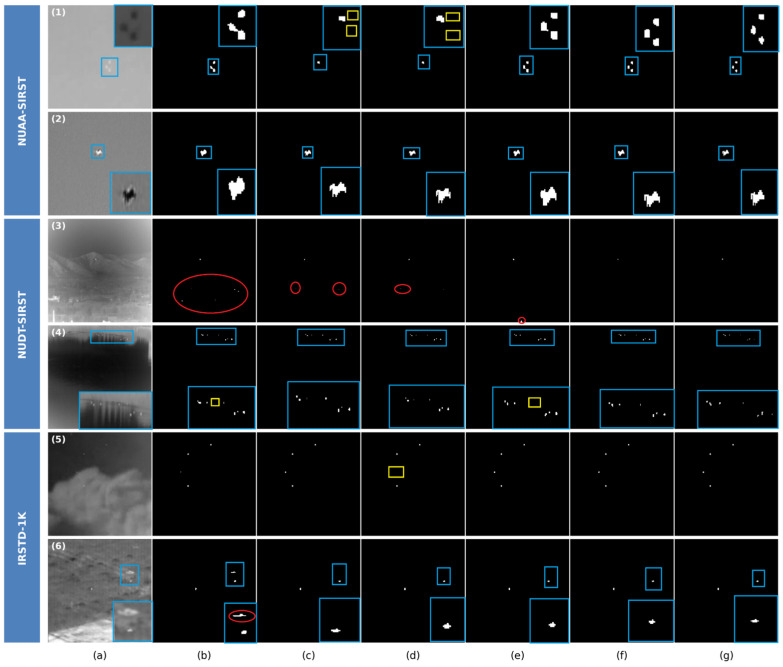
Visual results obtained using different IRSTD methods on the NUAA-SIRST, NUDT-SIRST, and IRSTD-1K datasets. Blue, yellow, and red represent correctly detected targets, missed detections, and false positives, respectively. (**a**) Input. (**b**) ACMNet. (**c**) DNANet. (**d**) UIU-Net. (**e**) SCTransNet. (**f**) CFGMNet. (**g**) GT, ground truth.

**Figure 9:**
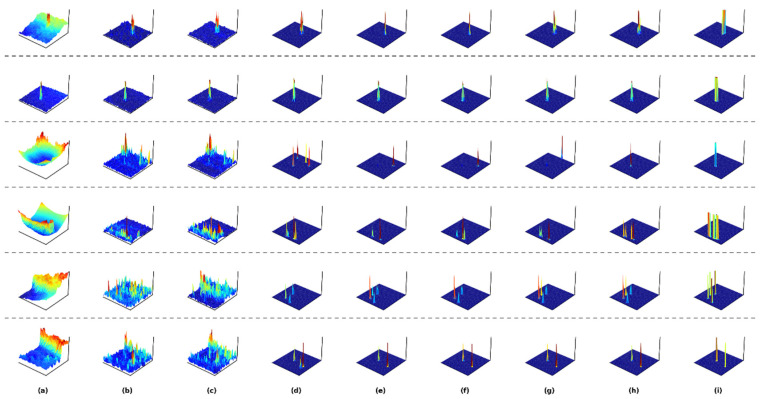
3D visualization of saliency maps generated by different methods on six test images. (**a**) Input. (**b**) Top-Hat. (**c**) TLLCM. (**d**) ACM. (**e**) DNANet. (**f**) UIU-Net. (**g**) SCTransNet. (**h**) CFGMNet. (**i**) GT, ground truth.

To provide a more intuitive comparison of the detection performance of different methods in complex scenarios, this paper presents pixel-level segmentation results and visualizations of feature responses, as shown in Figure 8 and Figure 9, respectively.

Figure 8 illustrates typical complex scenarios from the three datasets, including situations such as multiple object distributions, strong cluttered backgrounds, interference from building heat sources, small and faint targets, and low-contrast backgrounds. Specifically, red circles represent false positives, yellow boxes represent missed detections, and blue boxes represent correctly detected targets with local magnified details. Subfigures (1)–(2) are from NUAA-SIRST, (3)–(4) from NUDT-SIRST, and (5)–(6) from IRSTD-1K.

As can be seen from the segmentation results, traditional methods and some deep learning methods are prone to issues such as target truncation, jagged edges, target merging, or false background detections in complex backgrounds. For example, in scenes with strong clutter and heat source reflections, the comparison methods tend to misclassify locally bright areas as targets; in scenes with extremely small targets and low contrast, some methods suffer from missed detections or incomplete target regions. In contrast, CFGMNet effectively preserves the integrity of small target regions and significantly reduces false background responses, resulting in segmentation outcomes that align more closely with ground-truth annotations.

Figure 9 further illustrates the feature response distributions of different methods. The responses of traditional methods are relatively dispersed, with a significant amount of high-response noise in the background regions; while some deep learning methods have improved the intensity of target responses, they still suffer from residual background noise or the diffusion of target responses. In contrast, CFGMNet produces a more concentrated and prominent response peak in the true target region, while significantly suppressing the response in the background region. This phenomenon indicates that the proposed method can more effectively distinguish between small true targets and target-like background interference, consistent with the quantitative experimental results. Color mapping indicates saliency response intensity, where blue corresponds to low response and warm colors (yellow/red) correspond to high response.

**Figure 10:**
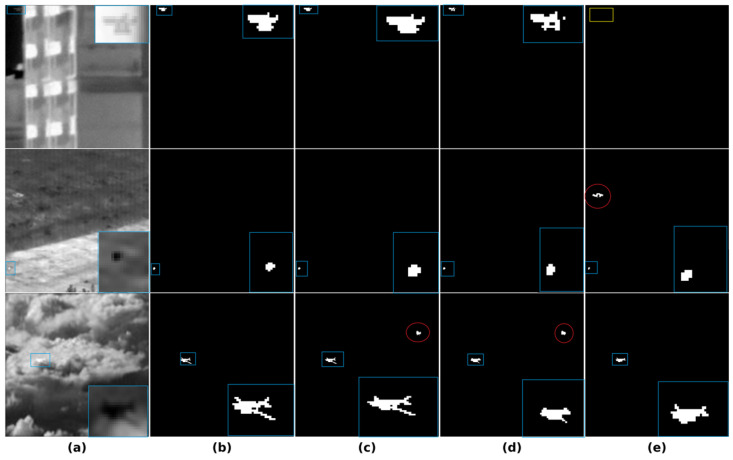
Representative failure cases on the NUDT-SIRST dataset, including blurred target boundaries, substantial target-scale variation, and weak target–background frequency differences. (**a**) Input image. (**b**) Ground truth. (**c**) SCTransNet. (**d**) UIU-Net. (**e**) CFGMNet.

To further analyze the performance differences on the NUDT-SIRST dataset, representative failure cases are presented in Figure 10. Red circles, yellow boxes and blue boxes denote false positives, missed detections and correctly detected targets, respectively.

As shown in Figure 10, SCTransNet and UIU-Net preserve relatively complete target responses in some challenging cases, although they may still produce false alarms in complex background regions. In contrast, CFGMNet suppresses background interference more effectively, but it may generate incomplete target responses or missed detections when target boundaries are blurred, target scales vary substantially, or target–background frequency differences are weak. This phenomenon indicates that CFGMNet adopts a relatively conservative response verification strategy. Such a strategy is beneficial for reducing target-like background false alarms, but it may also suppress weak or ambiguous true target responses. Therefore, the performance gap between CFGMNet and UIU-Net/SCTransNet on several NUDT-SIRST metrics reflects a trade-off between false-alarm suppression and target preservation. It also suggests that the current fixed-scale cross-frequency modeling remains limited in scenarios with large scale variation, blurred boundaries, and weak frequency contrast.

### 4.3. Model Complexity and Inference Efficiency

In addition to detection accuracy, a model’s computational complexity and inference speed are also critical factors in determining whether an infrared small target detection algorithm can be practically deployed. To evaluate the engineering potential of CFGMNet, this paper compares the number of parameters, computational cost, mIoU, and FPS of different methods on the NUAA-SIRST dataset; the results are shown in Table 4. Bold values indicate the best results, and underlined values indicate the second-best results. All models were tested on a single NVIDIA RTX 4090 GPU, with the input size uniformly set to 1 × 1 × 256 × 256 and a batch size of 1. During performance testing, all models were set to evaluation mode. Therefore, the reported FPS should be interpreted as an upper-bound estimate of model inference speed rather than end-to-end deployment throughput.

**Figure 11:**
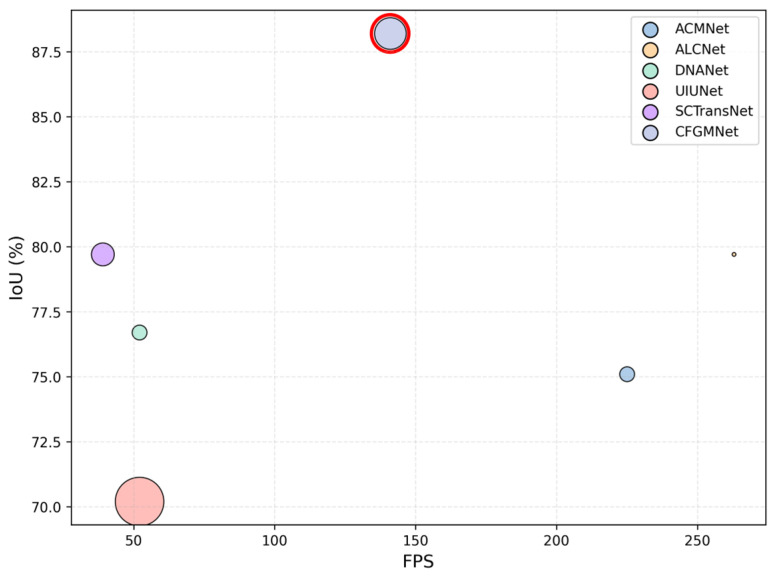
Comparison of the accuracy-speed trade-off among different methods. The red square represents the proposed CFGMNet, which achieves a favorable balance between detection accuracy and inference speed.

In terms of parameters and computational complexity, CFGMNet has 21.40 million parameters and 36.50 GFLOPs, which is higher than SCTransNet but significantly lower than ABCNet’s 106.99 million parameters and 166.27 GFLOPs. This indicates that our method enhances feature modeling capabilities while keeping model complexity within a reasonable range. In terms of inference speed, as shown in Figure 11, different methods exhibit a clear trade-off between accuracy and speed. Although ACMNet achieves the highest speed of 232 FPS, its mIoU is only 68.02%, indicating low detection accuracy; DNANet, ABCNet, and SCTransNet achieve FPS values of 16, 18, and 39, respectively. In contrast, CFGMNet achieves the highest mIoU while reaching 141 FPS under the same forward-pass evaluation protocol, approximately 3.62 times faster than SCTransNet. These results indicate that CFGMNet provides a favorable accuracy–efficiency trade-off under controlled GPU inference conditions.

These results indicate that CFGMNet achieves a good balance between accuracy and efficiency, improving segmentation accuracy while maintaining high inference speed. This is primarily due to Mamba’s linear-complexity modeling capabilities and the relatively lightweight architectural design of its auxiliary modules.

### 4.4. Robustness Analysis Under False Alarm Constraints and Low-SNR Conditions

**Figure 12:**
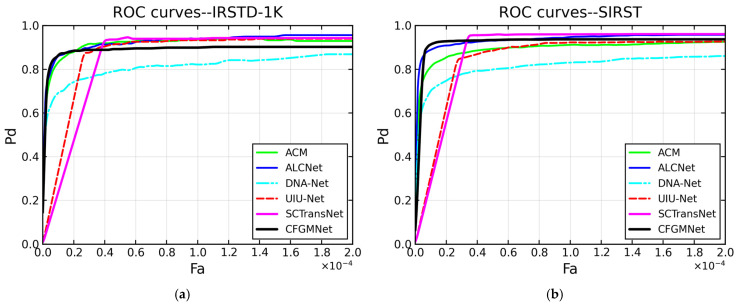
ROC curves of all compared methods on two infrared small target detection datasets. (**a**) ROC curves on the IRSTD-1K dataset, which compares the detection probability (Pd) against false alarm rate (Fa) across different methods; (**b**) ROC curves on the SIRST-3 dataset, presenting the corresponding Pd-Fa performance comparison of all involved algorithms.

To further evaluate the detection performance of different methods under various false alarm rate conditions, this paper plots ROC curves on relevant datasets, as shown in Figure 12. The closer the ROC curve is to the top-left corner, the higher the detection probability the model can achieve at the same false alarm rate, indicating superior overall performance. This metric is of great significance for practical applications with strict false alarm rate requirements, such as infrared early warning and maritime and aerial surveillance.

As shown in Figure 12, the ROC curve of CFGMNet generally lies above that of the comparison methods; in particular, it maintains a high detection probability even in the low false alarm rate region, indicating that our method effectively suppresses background false positives while preserving true target responses. In scenarios with complex backgrounds and weak targets, CFGMNet demonstrates more stable target-background discrimination capabilities compared to methods such as DNANet, UIU-Net, and SCTransNet, further validating the effectiveness of the cross-frequency gating mechanism, decoupled prediction heads, and hard negative sample optimization strategy for low-false-alarm detection.

**Figure 13:**
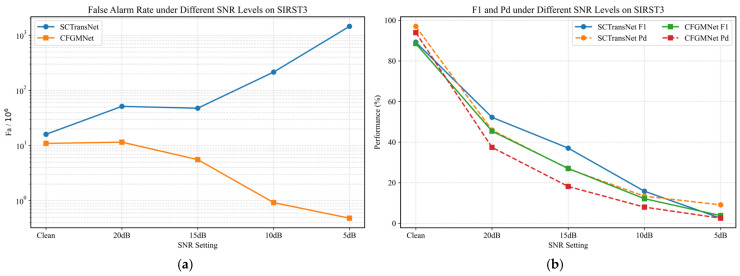
Robustness analysis under different SNR conditions on the SIRST3 dataset. (**a**) Trends of F1 and Pd. (**b**) Trend of Fa. Since Fa values vary greatly across different noise levels, the vertical axis in (**b**) is shown on a logarithmic scale.

Furthermore, to further analyze the model’s stability under noise interference, this paper adds random Gaussian noise of varying intensities to the SIRST3 dataset and compares the detection performance of SCTransNet and CFGMNet under different SNR conditions. The results are shown in Table 5 and Figure 13. Here, “Clean” refers to the original test images without added noise; a lower SNR indicates stronger noise interference.

Although the Pd of CFGMNet exhibits a decline under extreme noise conditions (e.g., 5 dB), its false alarm rate remains remarkably suppressed compared to baseline methods. In practical engineering applications, such as infrared guidance or maritime search and rescue, a conservative detection strategy that prioritizes an ultra-low false alarm rate is often more critical than blindly pursuing a high detection probability. Frequent false alarms caused by amplified noise can overwhelm subsequent tracking systems and lead to catastrophic system-level failures.

In contrast, CFGMNet consistently maintains a lower Fa across all noise levels: compared with SCTransNet, it reduces Fa by 31.73%, 77.56%, 88.32%, 99.57% and 99.97% under Clean, 20 dB, 15 dB, 10 dB and 5 dB conditions, respectively.

Comprehensive quantitative comparisons, visual analysis, performance evaluations, ROC curves, and noise interference experiments demonstrate that CFGMNet exhibits strong false alarm suppression capabilities and real-time inference potential in complex backgrounds. It should be noted that under extremely low SNR conditions, the F1 score and Pd of our method decrease significantly, although Fa remains very low. This indicates that the low false alarm performance of CFGMNet is partly attributed to a relatively conservative prediction strategy, and its target retention capability in high-noise scenarios still needs further improvement.

### 4.5. Ablation Study

#### 4.5.1. Structural Ablation

To validate the effectiveness of each core component of CFGMNet, this paper conducts structural ablation experiments on the NUAA-SIRST dataset. All experiments employ the same training parameters, data augmentation strategies, and testing environments, with the classic U-Net serving as the baseline model. Following a low-false-alarm detection approach of “global context screening—local saliency calibration—decoding noise filtering—final true/false classification,” this paper progressively introduces cross-frequency gating modules, local contrast gating, attention-based decoding mechanisms, and decoupled prediction heads. The experimental results are shown in Table 6.

As shown in Table 6, the baseline U-Net achieves a Pd of 96.33% but a high Fa of 32.08, indicating that the core challenge lies in false-alarm suppression rather than target detection. Adding the bottleneck CFG-Mamba reduces Fa by 47.0% and improves mIoU by 5.96 percentage points, confirming its dominant role in clutter suppression. Inserting an additional CFG-Mamba after the fourth encoder stage further lifts mIoU to 78.46% and reduces Fa to 13.95. Introducing LCG brings mIoU to 79.28% and Fa to 10.29, complementing global context filtering with local saliency constraints. Adding AG and CA improves mIoU to 82.53% and reduces Fa to 7.62, validating the effectiveness of suppressing noise introduced by skip connections. Finally, the decoupled prediction head further reduces Fa to 5.98 and raises mIoU to 85.27%, with Pd remaining at 99.08%.

Overall, these modules correspond to different stages of the proposed low-false-alarm pipeline. CFG-Mamba performs cross-frequency candidate screening, LCG conducts local saliency calibration, the residual attention decoder prevents skip connections from reintroducing background noise, and DH provides output-level response verification. Therefore, the performance gain comes from a progressive false-alarm suppression mechanism rather than from simply stacking multiple modules.

**Figure 14:**
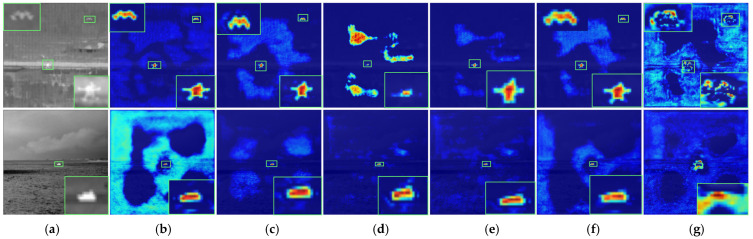
Visualization of the mid-layer feature responses for different ablation variants (**a**) shows the original image; (**b**) shows the full CFGMNet; (**c**–**g**) show the response results after removing the bottleneck layer CFG, X4CFG, LCG, AG + CA, and DH, respectively.

To further analyze the impact of each module on feature responses, this paper visualizes the responses of intermediate layers for different ablation variants, as shown in Figure 14. This figure uses the Jet colormap (blue to red = low to high response intensity); green boxes mark target locations, with insets showing magnified target details.

As can be seen, the responses of the complete model are more concentrated in the regions of the actual small targets, with weaker responses in the background regions; whereas after removing the relevant modules, the model is more likely to generate responses in the bright background regions, and the concentration of target responses also decreases. These results indicate that cross-frequency gating, local contrast gating, attention decoding, and decoupled prediction heads can work together to enhance target salience and suppress background interference. The feature visualization results are consistent with the quantitative results in Table 6, further validating the effectiveness of each module in reducing false positives and improving detection performance.

#### 4.5.2. Ablation Study of Loss Functions

To address the issues of low target pixel proportion and extreme imbalance between positive and negative samples in infrared small target detection, this paper conducts loss function ablation experiments on the full CFGMNet architecture. Starting with the baseline BCE loss, the experiments progressively introduce Dice Loss, OHEM, and Focal Loss to evaluate the impact of different loss components on segmentation accuracy and false alarm suppression. The results are shown in Table 7.

As shown in Table 7, vanilla BCE yields conservative predictions with low Fa but only 74.08% mIoU. Adding Dice Loss substantially improves mIoU to 82.95%. Introducing OHEM further lifts mIoU to 84.07% and reduces Fa to 6.82. The full composite loss with Focal Loss achieves the best overall performance, with 85.27% mIoU and 5.98 Fa.

#### 4.5.3. Receptive Field Analysis of LCG

To verify the impact of the local background estimation scale in the LCG module, this paper compares LCG designs with different receptive field sizes on the SIRST3 dataset; the results are shown in Table 8.

As shown in Table 8, all LCG variants outperform the baseline without LCG. The 3 × 3 window yields the highest Pd but the highest Fa; the 7 × 7 window achieves the lowest Fa but degrades Pd and mIoU. The 5 × 5 window achieves the optimal balance among Pd, Fa and mIoU, and is thus adopted in the final model.

#### 4.5.4. Sensitivity Analysis of Frequency-Decoupling Strategies

It should be noted that in this set of comparative experiments, only the low-frequency estimation operation within CFG-Mamba was replaced; the residual high-frequency computation, the Mamba-based low-frequency modeling branch, the spatial gating mechanism, the feature fusion layer, and all training settings remained unchanged. Therefore, this experiment is specifically designed to verify the sensitivity of the frequency decoupling strategy proposed in this paper to various low-frequency extraction operators.

As shown in Table 9, this module does not exhibit significant sensitivity to the average pooling kernel size. Among the options, the 3 × 3 average pooling scheme delivers the best overall performance, with the lowest false alarm rate and the highest mIoU and F1 scores. When the pooling kernel is expanded to 5 × 5 or 7 × 7, the false positive rate increases slightly, while segmentation accuracy does not improve. This indicates that excessive smoothing weakens effective local target candidate features and introduces more background residuals into the high-frequency branches.

The Gaussian filtering scheme achieved the highest detection rate (Pd), but its false alarm rate (Fa) also peaked among all compared schemes. This indicates that while low-pass filtering with a higher degree of smoothing preserves more target candidate regions, it is also more likely to retain and activate a large amount of background clutter that resembles the target.

For the wavelet decomposition experimental group, this paper uses only the LL subband of the Haar wavelet to reconstruct the low-frequency components: the LL subband represents the approximation component (background information), while the LH, HL, and HH subbands primarily correspond to high-frequency details in various directions. This approach maintains the same “low-pass extraction + residual high-frequency calculation” decomposition logic as average pooling and Gaussian filtering, meaning that the high-frequency response is still obtained by subtracting the reconstructed low-frequency component from the original features. The Haar wavelet LL scheme did not yield any performance improvement; this is most likely because a wavelet decomposition with a step size of 2 and reconstruction using only the LL subband results in the loss of the fine localization features required for detecting small targets.

Based on all the experimental results, we can conclude that CFG-Mamba’s performance advantage stems primarily from its cross-frequency context-guided verification mechanism, rather than from any specific low-pass operator. The simple 3 × 3 average pooling scheme requires no additional hyperparameters, incurs lower computational overhead, delivers stable performance, and achieves a good balance between target retention and false alarm suppression.

## 5. Discussion

The experimental results indicate that CFGMNet achieves a favorable balance between segmentation accuracy and false-alarm suppression. Its strong performance on NUAA-SIRST and low Fa on IRSTD-1K suggest that verifying high-frequency candidate responses under low-frequency background constraints is effective for suppressing target-like clutter, such as cloud edges, sea-surface reflections, building heat sources, and isolated bright noise.

Additionally, a systematic sensitivity analysis for the composite loss-function weights and the hard negative mining strategy was not conducted in this work. This hyperparameter configuration relies partly on empirical engineering experience, which stands as a limitation to be addressed in future research.

However, CFGMNet still has limitations. On NUDT-SIRST, some metrics are lower than those of UIU-Net and SCTransNet, which may be attributed to large target-scale variations, blurred boundaries, and weak frequency differences between targets and backgrounds. In such cases, the background-constrained verification strategy may suppress ambiguous true target responses together with false responses. Similarly, under severe low-SNR conditions, CFGMNet maintains a low false alarm rate but suffers from decreased Pd and F1, indicating a trade-off between false-alarm suppression and weak-target preservation.

Future work will focus on multi-scale cross-frequency modeling, adaptive local windows, bidirectional contrast estimation and dynamic gating strategies to improve target retention under multi-scale, low-SNR and contrast-reversed scenarios. Cross-sensor, cross-scene and platform-level validation, together with model pruning, quantization and embedded deployment optimization, will also be conducted to enhance the practical applicability of CFGMNet.

## 6. Conclusions

This paper proposes CFGMNet, a cross-frequency context-guided Mamba network designed for the detection of small infrared targets with low false alarm rates. By utilizing CFG-Mamba to model long-range low-frequency background characteristics and filter high-frequency candidate regions, the method mitigates the issue of confusion between real targets and target-like clutter in complex backgrounds.

Future research will focus on multi-scale, cross-frequency modeling, adaptive gating mechanisms, and lightweight deployment methods to enhance the model’s robustness and practical value in scenarios with low signal-to-noise ratios, multi-scale targets, and real-world embedded environments.

## Figures and Tables

**Table 1:** Conceptual comparison between CFGMNet and representative IRSTD methods. The comparison focuses on backbone type, core idea, and the key methodological difference from CFGMNet.

Method	Backbone Type	Core Idea	Key Difference from CFGMNet
SCTransNet	Transformer	Spatial–channel attention	No explicit frequency decomposition
MiM-ISTD	Mamba	Global–local Mamba modeling	Lacks background-constrained verification
IRMamba	Mamba	Difference-enhanced Mamba	No structured frequency gating
SAMamba	Mamba	Hierarchical state-space fusion	No low/high-frequency separation
EAMNet	Mamba-based	Feature enhancement	Weak false-alarm suppression
CFGMNet	Mamba-CNN hybrid	Frequency-guided modeling with background-aware gating	Explicit low–high frequency decomposition with background-aware gated verification

**Table 2:** Quantitative comparison with representative IRSTD methods on three benchmark datasets. Pd, F1, mIoU, and nIoU are reported in %, while Fa is reported in ×10^−6^. ↑/↓ indicates that higher/lower values are better. Bold and underline denote the best and second-best results, respectively.

Method	NUAA-SIRST					NUDT-SIRST					IRSTD-1K				
	Pd ↑	Fa ↓	F1 ↑	mIoU	nIoU	Pd ↑	Fa ↓	F1	mIoU	nIoU	Pd ↑	Fa ↓	F1 ↑	mIoU	nIoU
Top-Hat [6]	79.84	1012	14.63	7.14	18.27	78.41	166.7	33.52	20.72	28.98	75.11	1432	16.02	10.06	7.438
TLLCM [7]	79.09	5899	5.00	1.03	4.10	62.01	1608	7.23	2.18	4.32	77.39	6738	2.19	3.31	0.78
Max-Median [10]	69.20	55.33	10.67	4.17	12.31	58.41	36.89	7.64	4.20	3.67	65.21	59.73	8.15	7.00	3.05
WSLCM [8]	77.95	5446	4.81	1.16	6.84	56.82	1309	5.99	2.28	3.87	72.44	6619	2.13	3.45	0.68
ACM [12]	90.63	16.23	80.87	68.02	68.67	91.64	58.71	76.40	61.81	66.25	**92.49**	56.07	74.25	62.17	58.18
ALCNet [15]	93.20	39.15	83.92	71.53	70.25	93.35	37.42	79.60	65.83	69.20	92.25	59.80	75.75	61.28	56.14
DNANet [13]	94.50	9.78	86.83	76.73	80.52	96.61	9.87	92.14	87.74	89.74	90.92	13.72	78.62	63.42	65.71
UIU-Net [14]	95.74	15.86	86.93	76.58	78.93	**98.79**	10.79	96.29	93.84	93.81	93.23	22.70	76.67	64.99	65.81
SCTransNet [4]	97.24	14.67	89.10	80.32	83.60	98.51	**4.29**	**96.95**	**94.21**	**94.23**	92.27	10.74	79.72	66.30	66.39
EAMNet [27]	96.25	**4.94**	83.69	76.28	74.59	90.62	26.14	79.52	65.32	70.28	90.91	27.05	75.36	63.28	57.13
CFGMNet	**99.08**	5.98	**93.77**	**85.27**	**86.09**	95.87	9.32	94.29	89.18	90.02	88.89	**9.11**	**79.83**	**66.62**	**67.02**

**Table 3:** Statistical stability analysis of CFGMNet under different random seeds. Results are reported as mean ± standard deviation over three independent runs with seeds 42, 123, and 456. Best results are not cherry-picked. All experiments follow identical training settings. ↑ and ↓ indicate that higher values and lower values are better, respectively.

Metric	SIRST3	NUAA-SIRST	NUDT-SIRST	IRSTD-1K
mIoU (%) ↑	79.38 ± 0.22	85.25 ± 0.90	89.23 ± 0.06	66.48 ± 0.12
nIoU (%) ↑	82.64 ± 0.19	86.37 ± 1.14	90.02 ± 0.08	67.39 ± 0.50
Pd (%) ↑	94.48 ± 0.47	99.69 ± 0.53	95.94 ± 0.42	90.69 ± 1.59
Fa (×10^−6^) ↓	11.86 ± 0.82	5.86 ± 0.20	9.49 ± 0.15	9.14 ± 1.22
F1 (%) ↑	88.52 ± 0.14	93.19 ± 0.53	94.12 ± 0.29	79.34 ± 0.42

**Table 4:** Comparison of model complexity, accuracy, and inference speed on NUAA-SIRST. Parameters are reported in M, FLOPs in G, mIoU in %, and FPS under the same forward-pass-only evaluation protocol.

Method	Type	Param (M)	FLOPs (G)	mIoU	FPS
ACMNet	CNN	**0.52**	**1.01**	68.02	**232**
DNANet	CNN	4.7	28.56	76.73	16
ABCNet	CNN + ViT	106.99	166.27	81.01	18
SCTransNet	CNN + ViT	11.19	10.17	80.32	39
CFGMNet	CNN + Mamba	21.4	36.5	**85.27**	141

**Table 5:** Robustness comparison under different SNR conditions on SIRST3. Clean denotes original test images without added Gaussian noise. F1 and Pd are reported in %, while Fa is reported in ×10^−6^.

Model	Setting	F1	Pd	Fa
SCTransNet	Clean	89.21	97.01	15.98
CFGMNet	Clean	88.57	93.95	10.91
SCTransNet	20 dB	52.21	45.91	51.28
CFGMNet	20 dB	45.41	37.41	11.51
SCTransNet	15 dB	37.03	26.84	47.52
CFGMNet	15 dB	27.02	18.14	5.55
SCTransNet	10 dB	15.80	13.36	214.94
CFGMNet	10 dB	12.12	7.97	0.92
SCTransNet	5 dB	2.51	9.10	1451.77
CFGMNet	5 dB	3.80	2.52	0.48

**Table 6:** Structural ablation study. CFG denotes the CFG-Mamba module at the bottleneck stage, X4CFG denotes the CFG-Mamba module inserted after the fourth encoder stage, LCG denotes the Local Contrast Gate, AG denotes the spatial attention gate, CA denotes channel attention, and DH denotes the decoupled prediction head. ✔ denotes the module is adopted, and × denotes the module is removed.

Method	CFG	X4CFG	LCG	AG	CA	DH	mIoU	nIoU	Pd	Fa
Baseline	×	×	×	×	×	×	69.34	69.17	96.33	32.08
I	✔	×	×	×	×	×	75.30	77.26	97.25	15.03
II	✔	✔	×	×	×	×	78.46	79.17	97.25	13.95
III	✔	✔	✔	×	×	×	79.28	82.24	99.08	10.29
IV	✔	✔	✔	✔	✔	×	82.53	84.25	99.08	7.62
Full model	✔	✔	✔	✔	✔	✔	85.27	86.09	99.08	5.98

**Table 7:** Loss function ablation study. ✔ denotes the module is adopted, and × denotes the module is removed. Bold values indicate the best results, and underlined values indicate the second-best results.

BCE	Dice	OHEM	Focal	Pd	Fa	mIoU
✔	×	×	×	98.17	**5.11**	74.08
✔	✔	×	×	**99.08**	7.33	82.95
✔	✔	✔	×	**99.08**	6.82	84.07
✔	✔	✔	✔	**99.08**	5.98	**85.27**

**Table 8:** Ablation study of LCG module with different receptive field sizes. Bold values indicate the best results, and underlined values indicate the second-best results.

Method	Pd	Fa	mIoU
3 × 3-LCG	93.40	12.12	78.63
5 × 5-LCG	**93.95**	10.35	**79.48**
7 × 7-LCG	92.78	**9.98**	77.82
Without LCG	91.96	12.29	76.79

**Table 9:** Performance comparison of different frequency decomposition strategies on the SIRST3 dataset. ↑ and ↓ indicate that higher values and lower values are better, respectively.

Method	Pd ↑	Fa ↓	mIoU ↑	F1 ↑
3 × 3 AvgPool	93.95	10.35	79.43	88.57
5 × 5 AvgPool	94.15	11.75	78.88	88.19
7 × 7 AvgPool	93.89	12.51	78.88	88.19
Gaussian (σ = 1.0)	94.68	13.01	78.95	88.24
Haar-LL	93.36	12.52	78.04	87.67

## Data Availability

The public datasets used in this study, including NUAA-SIRST, NUDT-SIRST, and IRSTD-1K, are available from their corresponding official repositories or publications.

## Abbreviations

The following abbreviations are used in this manuscript:

CFGMNet	Cross-Frequency Context-Guided Mamba Network
LCG	Local Contrast Gate
AG	Attention Gate
CA	Channel Attention
DH	Decoupled Head
GT	Ground Truth
ROC	Receiver Operating Characteristic
SNR	Signal-to-Noise Ratio
BCE	Binary Cross Entropy
OHEM	Online Hard Example Mining
IoU	Intersection-over-Union
